# From ‘Pen Sao’ to ‘Tue Pa’: Understanding diverse pathways to adolescent pregnancy in Lao People’s Democratic Republic through qualitative investigation with girls in Vientiane Capital, Vientiane Province, and Luang Namtha

**DOI:** 10.1371/journal.pgph.0002825

**Published:** 2024-02-02

**Authors:** Marie Habito, Julie Hennegan, Kiaosamphan Rasphone, Saysamone Phanthachith, Toulavan Sihanath, Maki Akiyama, Peter S. Azzopardi, Elissa Kennedy, Romyen Kosaikanont

**Affiliations:** 1 Global Adolescent Health Group, Burnet Institute, Melbourne, Victoria, Australia; 2 Centre for Adolescent Health, Murdoch Children’s Research Institute, Parkville, Victoria, Australia; 3 Melbourne School of Population and Global Health, University of Melbourne, Victoria, Australia; 4 Indochina Research Limited (Laos), Vientiane, Lao PDR; 5 UNFPA Asia Pacific Regional Office, Bangkok, Thailand; 6 Telethon Kids Institute, Adelaide, South Australia, Australia; 7 School of Public Health and Preventive Medicine, Monash University, Melbourne, Victoria, Australia; 8 School of Management, Mae Fah Luang University, Chiang Rai, Thailand; Queen’s University, CANADA

## Abstract

Adolescent birth rates in Lao People’s Democratic Republic (PDR) remain the highest in Southeast Asia. There is growing recognition that adolescent pregnancy in Lao PDR is occurring within and outside marriage, but there is a lack of robust qualitative evidence to understand girls’ pathways to adolescent pregnancy and contributing factors, especially outside of union (cohabitation or marriage). This study aimed to improve understanding of pathways to adolescent pregnancy in Lao PDR among girls who experienced pregnancy at age 18 or below. We conducted participatory timeline interviews with 57 girls from urban, peri-urban, and rural communities in Vientiane Capital, Vientiane Province, and Luang Namtha, and follow-up interviews with a subset of 20 girls. We identified six pathways to pregnancy, including pathways outside (n = 23) and within union (n = 34). Outside-union pathways diverged according to the nature of sex preceding pregnancy (consensual/pressured, or forced), and pregnancy intention (unplanned, partner-led, or planned). Within-union pathways diverged according to the nature of the relationship before union (romantic or no romantic relationship/arranged union), who initiated the union (couple/girl, parent/partner, or pressured), and pregnancy intention. Factors contributing to girls’ pregnancy included barriers to sexual and reproductive health (SRH) information and services; partner’s control over reproductive decision-making; male sexual entitlement and alcohol use driving pressured/forced sex; cultural acceptance of child marriage and early union; and attitudes and norms regarding sex and pregnancy outside of union. Our findings support strengthening comprehensive sexuality education, including a focus on addressing myths about contraception, building girls’ and boys’ communication skills, engaging in respectful relationships, and addressing harmful gender norms. Our findings also highlight the need to improve girls’ access to adolescent-responsive SRH services, address harmful substance use, challenge sociocultural barriers to young people accessing SRH information and services, and respond to sociocultural and financial drivers of child marriage/early union that contribute to adolescent pregnancy.

## Introduction

Lao People’s Democratic Republic (PDR) is home to roughly 1.5 million adolescents aged 10–19 years, half of whom are adolescent girls [1]. Over the last two decades, Lao PDR has consistently had the highest adolescent birth rate (ABR) in Southeast Asia. Currently, the ABR in Lao PDR is 72 births per 1,000 girls aged 15–19 years [2], far exceeding the regional average for Asia-Pacific of 21 births per 1,000 girls aged 15–19 years [1, 3]. The birth rate varies between rural and urban areas, reaching as high as 136 births per 1,000 girls aged 15–19 years in rural areas without roads [4]. Consequently, maternal and neonatal complications remain among the leading causes of death among female adolescents aged 15–19 years in Lao PDR [5].

At the 25^th^ anniversary of the International Conference on Population and Development (ICPD25), the Government of Lao PDR committed to ending maternal mortality, addressing unmet need for family planning among adolescent girls, reducing child marriage by 2030, and fully integrating comprehensive sexuality education in national school curricula [6, 7]. In line with these commitments and the Sustainable Development Goals, the ‘Noi Framework’ was launched in 2016 –a roadmap aimed at raising awareness and fostering multisectoral partnership and investment to address challenges and empower adolescent Lao girls [6, 8]. While gains have been made since 2016, reduction of the ABR has been slow [2].

Adolescent pregnancy is closely linked to child marriage and early union in Lao PDR, particularly in ethnic communities where child marriage is considered the norm [4, 9, 10]. Among Lao women aged 20–24 years, one-third were married, and 18.4% had a livebirth before age 18 [4, 11]. Though prior research highlights adolescent pregnancy as an outcome of child marriage and early union [4, 9], there is growing recognition that adolescent pregnancy also occurs outside marriage [12, 13]. Recent analysis of Demographic and Health Surveys (DHS) data found that among Lao women aged 20–24 years who had ever been in a union and gave birth before they were 18 years old, 27% conceived before marriage or cohabitation, and premarital conception rates increased by two-thirds in the last five years [14].

A range of socio-demographic risk factors for adolescent pregnancy have been reported in Lao PDR, including residence (i.e., urban, rural, rural-off-road). Data from the 2017 Lao Social Indicator Survey show that adolescent births are more common among young women who are from rural areas, have lower level of education, and belong to lower wealth quintiles [4]. Adolescent births are also more common among young women from Hmong-Mien and Chinese-Tibetan ethnolinguistic backgrounds relative to those from Lao-Tai and Mon-Khmer backgrounds [4]. Studies have also highlighted that parent-child communication about sexual and reproductive health (SRH) is uncommon [15], pregnancy health literacy among adolescents is low [16], and young people face multiple barriers to SRH services [17]. These factors suggest that Lao girls’ trajectories to adolescent pregnancy are varied and complex. Yet, there is limited research capturing young Lao women’s lived realities of pregnancy [18, 19], which should be the basis of designing and implementing programmes and policies.

This study is part of a larger qualitative investigation to improve understanding of the pathways to and drivers of adolescent pregnancy in four countries in Southeast Asia. In this paper, we report on the findings from the research undertaken in Lao PDR. Specifically, we aimed to identify pathways to adolescent pregnancy from ‘*Pen Sao*’ (becoming a teenager) to ‘*Tue Pa’* (getting pregnant), salient features of each pathway, and crosscutting factors contributing to adolescent pregnancy that are found across pathways.

## Methods

This study is reported according to the Standards for Reporting Qualitative Research guidance [20].

### Study setting

A Working Group comprised of focal points from UNFPA and UNICEF country offices, two youth advisors (females aged 18–24), and two government representatives from the Ministry of Planning and Investment was established to review methodology and study materials, provide guidance on site selection, and support interpretation and dissemination of findings. Site selection was done in consultation with the Working Group, and informed by UNFPA internal analysis of adolescent fertility and estimated premarital conception using DHS data for each province, along with cost and feasibility constraints.

Vientiane Capital and Vientiane Province were selected to provide urban and rural sites with median levels of adolescent fertility and premarital conception (i.e., close to national average rates), while urban and rural sites in Luang Namtha were selected to represent a high level of adolescent fertility and premarital conception. Within each province, site areas were selected with input from the Working Group with the aim of representing the demographics of the district. Vientiane Capital (Prefecture) is the second most populous administrative region in the country and is home to almost one million people (population density of 247 people per km^2^) [21]. Vientiane Province is the fifth most populous administrative region in Lao PDR, with a population of 468,000 and occupies 15,610 km^2^ (population density of 30 people per km^2^) [21]. Vientiane Province contributes about 4% of the national gross domestic product largely from the service (including tourism) and industry sectors; however, in rural areas, agriculture remains the main source of livelihood. Luang Namtha, a predominantly agricultural province in the country’s northwest, is home to 202,000 people and occupies a total land area of 9,325 km^2^ (population density of 5 people per km^2^) [21].

### Ethical approvals

Ethical approval was provided by the Alfred Hospital Research Ethics Committee (ID: 14/21) and the Lao People’s Democratic Republic Ministry of Health National Ethics Committee for Health Research (NECHR) (ID: 2020.87.MC).

All participants provided written informed consent, including for audio recording, prior to timeline interview. Participants provided verbal consent for follow-up telephone interviews and were sent an electronic copy of the participant information sheet and notified that support organisations had hard copies of the information sheet available. Lao family law recognises young people aged 15–17 years as able to provide informed consent, and girls invited to participate in our study were pregnant or parenting at the time of interview and had already made decisions regarding their healthcare. Thus, girls recruited for the study were considered as having the evolving capacity and maturity to understand the study and provide informed consent to participate in research without the need for parental/guardian’s consent [22].

Two participants were 15 years old at interview. These were reported to the Alfred Hospital Ethics Committee and the in-country research team received immediate refresher training to reemphasise the need to clearly establish participant age prior to obtaining consent. The research team and ethics committee determined that the most ethical course of action was to retain the participants’ data in the study. The ethics committee recommended no further action.

### Recruitment

Adolescent girls aged between 16 and 20 years who had become pregnant at age 18 or younger were identified through partnership with social support organisations in each study area. Most rural participants were invited (in person) a day before interview, while urban participants were invited via phone call to participate in the study. We undertook rolling recruitment and recorded participant details against a sampling matrix, recruiting purposively to achieve diversity in rural-urban residence, ethnic background, and pre- and post-marital conception.

### Data collection 1: In-depth timeline interviews

Two female interviewers (KR, SP) received five days of training, including a pilot interview. The study youth advisors supported two days of training as paid consultants, participating in activities to inform the language used in the interview and interviewer practice engaging adolescents.

Interviews were conducted between March and August 2021. Interviews were done at a time and location convenient for participants (often in a private location on household grounds with auditory, and where possible, visual privacy) and lasted between 60 and 90 minutes. Participants were informed of their right not to discuss topics and to decline to answer any questions or end the interview. Discussions were paused if needed for childcare or other interruptions.

Interviews used a participatory, timeline approach and were supported by a semi-structured topic guide. Interviews commenced by asking participants to draw a timeline of their life story and the way that pregnancy and marriage fit into their story. In cases where participant literacy was low, the interviewer drew the timeline for the participant with their permission. The semi-structured topic guide was then used to explore girls’ timing of pregnancy and feelings about becoming pregnant; pregnancy intentions; relationship with the partner at the time of pregnancy; timing of union; experiences of learning about sex and reproduction, sex, and romantic relationships, and using contraception; other important family and community relationships; timeline and experience of schooling; and hopes and plans for the future. In moving through discussion topics, participants were asked if they wanted to place other experiences onto their timeline and timelines were used to clarify the temporality of events. Interviews were audio-recorded and transcribed into Lao by the interviewers (KR, SP), and timelines were photographed. Participants were assigned unique participant identification (ID) numbers (e.g., LA0101) and any personally identifiable information in the interview transcripts were replaced with generic alternatives (e.g., replaced names with ‘boyfriend/partner’, ‘mother’, ‘regional healthcare facility’). Only authorised members of the Lao PDR research team had access to participant contact information during data collection and solely for the purpose of recontacting participants who were willing to participate in follow-up interviews. Participant contact information was stored separately from study data and could not be linked to the participant ID.

### Analysis

We undertook framework analysis to describe, compare, and contrast the pathways to first adolescent pregnancy shared by participants [23]. Framework analysis is a thematic analysis approach which involves five steps: 1) familiarisation, 2) developing an initial analytical framework, 3) indexing (during which we summarised interviews according to the framework), 4) charting and summarising through development of a complete framework matrix, and 5) interpretation and mapping (during which cross-case analysis was undertaken to develop preliminary and final pathway typologies) [24].

For familiarisation, the study team undertook regular debriefing to share initial impressions from interviews. Lao-speaking team members developed initial themes. The analysis team (JH, MH, RK, KR, SP) then developed a comprehensive interview summary template capturing preliminary drivers and themes identified along with other temporal details. Team members fluent in Lao (RK, KR, SP) produced in-depth summaries of interviews in English and recorded key quotations for a diverse selection of 30 interviews. Through discussion, we developed a framework matrix to capture key temporal events and themes. Each participant’s data was entered into the matrix from English summaries by a Melbourne-based team member (JH) and by Lao team members directly from the remaining 27 transcripts, with the framework modified through discussion if needed to capture girls’ experiences.

A distinct component of framework analysis is the development of a framework matrix which enables data summary and display [24], and where each row corresponds to a case (a participant), and each column to a code (i.e., a theme or sub-theme, such as topics or issues observed across the dataset) [23, 25]. Consistent with the framework analysis approach, we used the framework matrix to undertake cross-case analysis [26] and identify pathway typologies. Preliminary pathways were discussed among the study team and presented to the study Working Group for feedback. We identified topic areas for validation and clarification with follow-up participants.

### Data collection 2: Follow-up telephone interviews

After preliminary analysis of the timeline interviews, we undertook follow-up interviews. These aimed to provide participant validation, clarification to preliminary findings, and to gather participant recommendations for policy and practice. Interviewers received a further three days of training on the follow-up content and to develop the videos, which was again supported by the youth advisors.

A subset of girls who provided permission and a phone number to be recontacted were called and invited to participate. Interviews were undertaken at a time nominated by the participant through a phone call. Four short and engaging videos and corresponding ‘radio-play’ audio recordings were developed to summarise key preliminary findings along topics of knowledge of sex and reproduction; contraceptive access and use; relationships and negotiating sex and pregnancy; and pregnancy and marriage. A semi-structured topic guide complemented the videos/audio recordings and explored girls’ impressions of the study findings, points of clarification on each topic, and recommendations for action around these topic areas. Videos were sent to girls with smartphones and audio recordings played back to girls with non-smartphones before interview. Interviews lasted no longer than 45 minutes and were paused if required. Interviewers made written notes during follow-up interviews and developed a written summary in English, after which all call and text history was deleted.

Insights from follow-up interviews were used to revise preliminary findings and pathways, and incorporated into written and presentation materials used during dissemination of study findings to stakeholders. Participant recommendations for policy and practice were analysed thematically and are reported elsewhere.

## Findings

**Fig 1:**
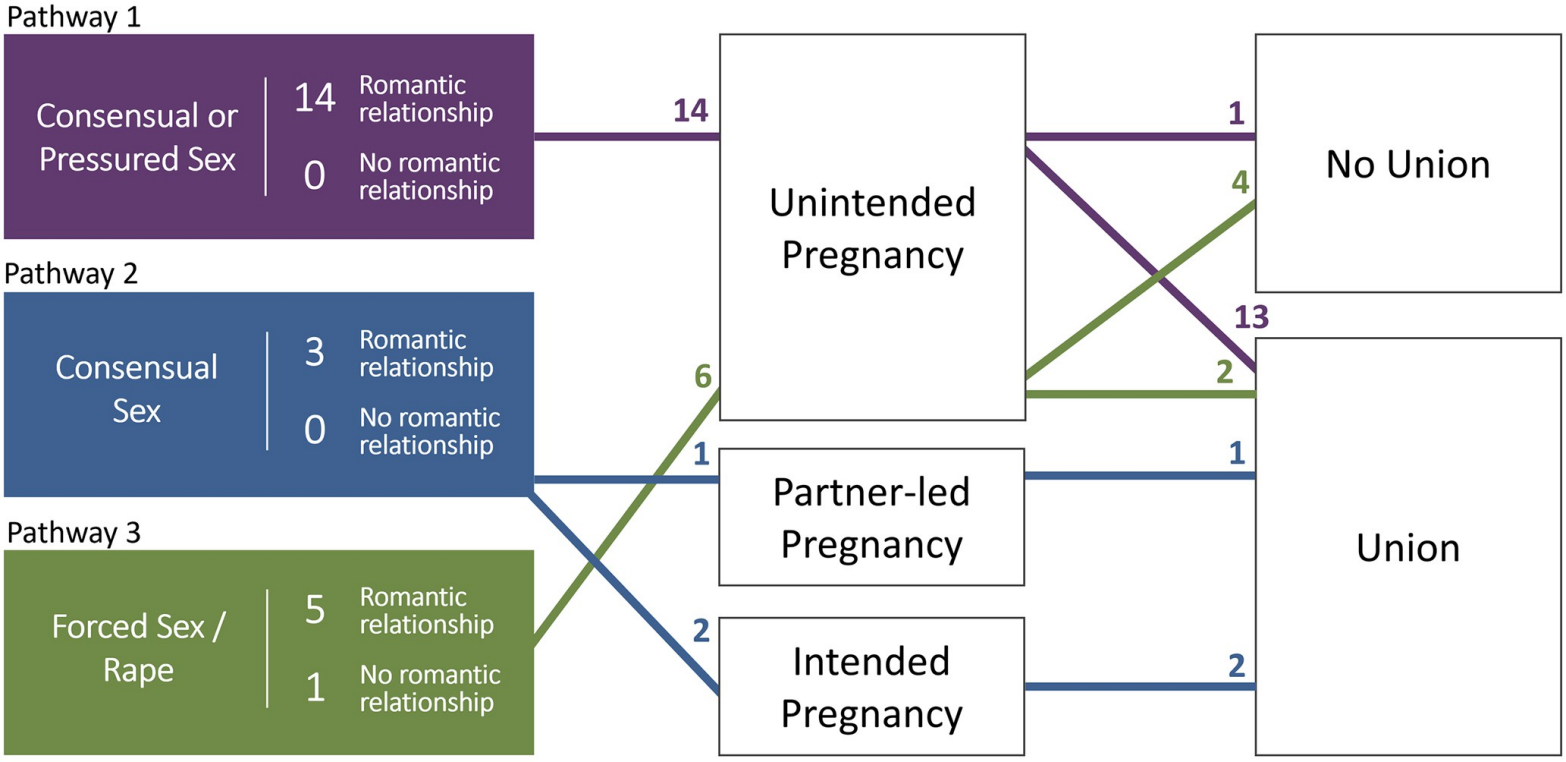
Pathways to adolescent pregnancy in Lao PDR, outside-union pregnancy pathways. Note: Roman numerals correspond to distinct pathways; figures near arrowheads refer to frequencies.

**Fig 2:**
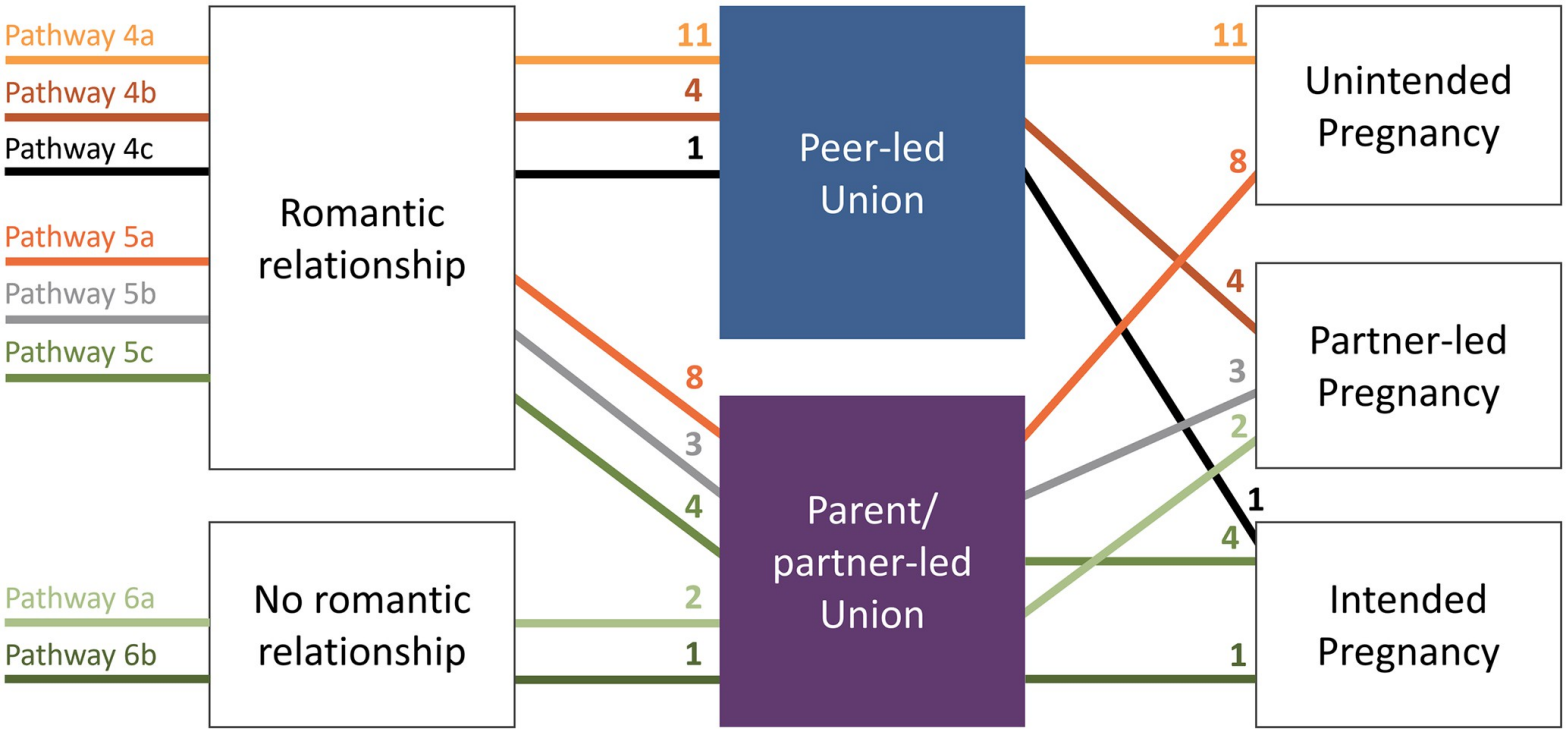
Pathways to adolescent pregnancy in Lao PDR, within-union pregnancy pathways. Note: Roman numerals correspond to distinct pathways; figures near arrowheads refer to frequencies.

We identified six pathways to pregnancy among our sample. Figs 1 and 2 provide visual representations of these pathways and the number of participants aligning with each. In developing the pathways, girls who became pregnant outside union were inductively grouped together, as were girls who became pregnant within union. Fig 1 illustrates the pathways of 23 girls who conceived outside of union–some before a subsequent cohabitation or marriage, and a few without ever entering a union. Identified pathways were consistent with prior analysis showing high levels of premarital conception in the Southeast Asian region [14]. Outside-union pathways were further differentiated by the nature of sex preceding pregnancy (consensual/pressured, or forced sex), and pregnancy intention (unplanned, partner-led, or planned pregnancy). Fig 2 illustrates the pathways of 34 girls who conceived within union. Their pathways to adolescent pregnancy were further differentiated by the nature of the relationship before union formation (romantic relationship or no romantic relationship/arranged union), who initiated the union (couple/girl-led, parent/partner-led, or pressured), and pregnancy intention (unplanned, partner-led, or planned pregnancy).

### Profile of participants

Fifty-seven interviews were undertaken between March and August 2021 with young women aged 15–20 years; the median age was 18 years. Eight participants were from Vientiane Capital (all urban), 16 from Vientiane Province (6 urban, 10 peri-urban), and 31 from Luang Namtha (16 urban, 15 rural). Definitions or ‘urban’ and ‘rural’ villages were based on the 2015 Lao Population and Housing Census [27], while the definition of a ‘peri-urban’ village is that used by the research partner organisation.

Twenty participants were married prior to becoming pregnant, 14 became pregnant after they started cohabitating with their partner but were not yet married, and 23 became pregnant prior to cohabitation or marriage. All participants had conceived their first pregnancy prior to age 18; the youngest at age 13, with a median age of 16 years. In Vientiane Capital and Province, almost all participants were of Lao Loum (‘lowland Lao’) ethnic background. In Luang Namtha, a diverse set of ethnicities were represented including Khamu, Hmong, Lantan, Lamet, Tai Dam, Lue, Phounoi, Sila, and Yung. Most participants had completed lower secondary school, while one-third had attended some lower secondary school but not completed. Four participants were secondary school graduates.

### Outside-union pregnancy pathways

For 23 girls, their pathway to pregnancy unfolded outside of union (cohabitation or marriage). Across the sample, girls began engaging in romantic relationships between ages 10 and 16. Many girls shared a similar story to that shared by LA0213: *‘The boy asked for my number from my friends*. *… He started chatting with me via WhatsApp*. *After six months of chatting*, *I felt that I like him*. *He then asked me out for* Pai Len *(party-going*, *usually involving alcohol consumption)*. *I said I would go if my friends would come along*. *So*, *we went out drinking beer with a group of friends*.*’* Most girls who got pregnant outside of union had other boyfriends before the partner who got them pregnant. Prior relationships usually involved text messaging and did not involve physical intimacy. Girls typically met the partner who got them pregnant through common spaces (e.g., school, work) and chatted through social media platforms before spending time together in person.

#### 1. Romantic relationship leading to consensual and ‘pressured’ sex and unplanned pregnancy

Within our sample, the most common pathway to adolescent pregnancy outside of union was through consensual or ‘pressured’ sex within a romantic relationship that led to unplanned pregnancy, followed by marriage or cohabitation in all except one case. Distinct in this pathway was girls’ description of having some say in the decision to have sex (e.g., they agreed to have sex), where some girls described sex as consensual, while others described being convinced to have sex following varying scenarios of sexual negotiation (referred to here as ‘pressured’ sex), characterised by emotional pressure and/or alcohol consumption. This pathway was observed across the three study areas, and among girls from urban, peri-urban, and rural communities. Girls were of Lao Loum, Khamu, Tai Dam, Phounoi, and Hmong ethnic backgrounds.

All girls in this pathway experienced sexual debut outside union, usually with the partner who got them pregnant; only two experienced sexual debut with a different person. ‘*Pai Len’* and alcohol consumption were salient in many narratives. Most girls described agreeing to their first sex with the partner who got them pregnant following some sexual negotiation which often occurred after the couple had been drinking at home or special events such as birthday parties:

*‘We were in the party and the music was loud*. *He talked to me saying that he would like to have “it”*. *I said “No*!*” … But then I do not know why I gave it to him*. *Maybe because I was drunk*.*’*

*(LA0233)*

Girls were concerned about pregnancy, and in some cases, the boyfriend’s promise that he would take responsibility for any pregnancy was central in girls’ decision making about whether to have sex.

Most girls in this pathway seemed to have limited understanding about sexual and reproductive health, and learned about contraceptives from peers; less than half reported receiving any sex education at school. Others received vague messages to ‘be careful’ from other people:

*‘The shopkeepers in the market and many people*, *especially senior [older] friends I know*, *they all told me to “be careful*, *don’t go out often*, *don’t focus on boys*.*” … They said if we are staying out late and playing around*, *we will have a baby*. *But I don’t really understand what they mean*.*’*

*(LA0218)*

Some girls indicated that they were aware of contraceptives but faced barriers to accessing them. For example, one girl knew about emergency contraceptive pills but felt too embarrassed to purchase them. Another girl reported that she and her partner agreed to use condoms, but she was too embarrassed to buy them; instead, they used the rhythm method but she miscounted and conceived. Eight girls reported using condoms during the first or second time they had sex with their boyfriend, but condom use was discontinued shortly after because the boyfriend did not like it or refused to continue using condoms. Some boyfriends promised to take responsibility for any pregnancy so the girl would agree to have unprotected sex. Four further girls described not attempting or discontinuing contraceptive use because they (or their partner) underestimated the risk of pregnancy following previous unprotected sex that did not result in pregnancy:

*‘We had sex the first time unprotected*. *But I was not pregnant*. *But we had sex a few times after that [was] protected*. *It was OK*. *But the last time we had sex*, *it was unprotected*, *then I became pregnant*.*’*

*(LA0202)*

All pregnancies were unplanned. After discovering the pregnancy, ten girls considered abortion even though abortion is illegal in Lao PDR with few exceptions [28]. Many were encouraged by friends to seek abortion. One girl terminated her first pregnancy, two attempted abortion with traditional herbs or taking pain killers or energy drinks mixed with soda, and another girl sought abortion care at a clinic but was refused. For other girls, their boyfriends’ or parents’ support for the pregnancy dissuaded abortion attempts:

*‘I already had an appointment with the doctor for abortion*. *Then my mother-in-law knew about it and cried so hard*. *She said that we only have a son*. *She said she wants more people at home*. *She begged me not to have [an] abortion*.*’*

*(LA0206)*

There were indications from a few girls’ stories that once parents became resigned to or accepted the pregnancy, the girl was well supported, and in one case, welcomed.

Almost all girls married or started living with the partner who got them pregnant within six months of the pregnancy. It was clear that parents expected marriage following pregnancy, but for three girls, cohabitation with the promise of marriage was accepted. One girl was living with her partner because his parents did not want the couple to marry. Union was more often parent-led, with some parents asking the boyfriend to take responsibility, or agreeing to marriage with the boyfriend’s parents. In four cases, union was peer-led (initiated by the couple or the girl). Most marriages were arranged through ‘*Pai Khor’*, a process whereby a marriage proposal is initiated by a young man and/or his family, and an approximate date for marriage is set with the girl’s family. Motivations for union included avoiding gossip and shame, avoiding fines for premarital pregnancy (customary among the Khamu), and ensuring economic security (i.e., to gain male labour for the girl’s family, to ensure the girl and her baby are provided for). Only one girl reported getting married for love. In a unique case, a girl did not enter a union because her parents did not want her to marry her boyfriend.

More than half of the girls in this pathway dropped out of high school due to their pregnancy. Others had stopping going to school for other reasons before becoming pregnant (e.g., parents wanted the girl to marry instead, girl fell behind due to poor health, financial reasons); one continued her education with support from parents.

#### 2. Planned or partner-led pregnancy to facilitate union (3)

Three participants’ narratives reflected a distinct pathway where pregnancy was intended to facilitate union. Girls in this pathway were from urban areas of Vientiane Province and Luang Namtha and were from Lantan (2) and Lao Loum (1) ethnic backgrounds.

Two girls were involved in deciding to become pregnant to facilitate cohabitation and/or marriage. The couples’ families were aware of the relationship but the girls had been encouraged to delay marriage and pregnancy. One girl’s parents wanted her to continue school and the other girl’s parents wanted her to spend more time getting to know her partner, but both were intent on pregnancy and union because of love. For the latter girl, her view that she was a financial burden on her family and community expectations of early marriage were also motivators. The couples were delighted by the pregnancy, and happy to be building their family:

‘[My partner] is excited and glad because he has always wanted to get married.’

*(LA0215)*

For the final participant, pregnancy was initiated by her partner. She described that they had agreed to wait until after she graduated from school to marry, but suspected her partner planned to impregnate her to speed their marriage. Following pregnancy, all three girls began living with their partners and became engaged.

The girls in this pathway learned about sex and contraception from female family members (e.g., sister, mother), friends, and schoolteachers. The girls who had planned pregnancies both used condoms during first sex then discontinued to become pregnant. The girl who had a partner-led pregnancy did not use any contraception. She had previously asked her partner to buy pills for her, but he refused. He similarly denied her abortion, and the couple’s parents agreed to their marriage.

#### 3. Forced sex/rape preceding unplanned pregnancy (6)

The third pathway to pregnancy outside of union was through forced sex (rape), usually perpetrated by the girl’s boyfriend. This pathway was observed in urban (5) and rural (1) areas from all three study sites, and from girls who belonged to Lao Loum (4), Khamu (1), and Lantan (1) ethnic backgrounds.

Five girls were raped by their boyfriend/partner, while one girl was raped by a friend’s boyfriend. For girls who were in romantic relationships, rape occurred at variable lengths into the relationship, from a few weeks to months. Among these, one girl had run away from home and was cohabitating with her boyfriend when she was raped by her boyfriend. Girls usually reported being under the influence of alcohol or other drugs, or unconscious during first sex; three girls had no recollection of the rape and only learned they had been raped when they woke up the following morning. Special events and ‘*Pai Len’* were again observed in this pathway, with some girls staying over at the boyfriend’s house, a friend’s house, or guesthouse following an event or drinking session.

None of the perpetrators of rape used condoms. One girl was told by her boyfriend about emergency contraceptive pills after she was raped, but she expected him to provide them and he did not.

Abortion was considered in all participant narratives. Three girls attempted abortion, but only one successfully terminated her pregnancy; the other two girls sought services at clinics but were dissuaded:

*‘I asked the doctor for where I can have abortion*. *The doctor said there is no such place and told me to ask for permission from my parents*.*’*

*(LA0231)*

Five girls were single at the time of the interview–four never entered a union with the partner who got them pregnant due to varied circumstances. Some girls had little choice, for example the partner’s parents refused the marriage:

*‘He said it is not his baby*. *But during the pregnancy*, *I moved to his mother’s home*. *They took care of me but when the baby was born*, *they did not allow me to marry him*.*’*

*(LA0240)*

Other girls had more say in the decision to not marry. For example, one girl refused to marry the partner who raped her and got her pregnant even though her parents were encouraging her to marry him; she felt the marriage would not last. The fifth single girl had a successful abortion after ending her relationship when it was clear that her boyfriend was not going to take responsibility for the pregnancy. One girl was forced by her father to marry the man who raped her:

*‘When I found out that I was pregnant*, *the father of the baby said it is not his*. *I was really upset and did not want anything to do with him anymore*. *But then my dad said I had to marry him because nobody at home will look after me*.*’*

*(LA0213)*

### Within-union pregnancy pathways

For 34 girls, adolescent pregnancy occurred in the context of child marriage and early union. Their pathways to pregnancy varied according to their relationship context prior to union, who initiated the union, and pregnancy intention.

#### 4. Romantic relationship leading to peer-led union and pregnancy

For 16 girls, romantic relationships followed by peer-led cohabitation or marriage resulted in either unintended (Pathway 4a), partner-led (Pathway 4b), or intended pregnancies (Pathway 4c). This pathway was observed in all three study areas across urban, peri-urban and rural communities, and girls in this pathway were of Lao Loum, Khamu, and Hmong ethnic backgrounds.

Girls in this pathway described the decision to cohabitate or marry as peer-led, where the boyfriend’s proposed union and the girl accepted. The main motivations for union were love and/or premarital sex. For example, in line with Hmong practice, one girl eloped with her boyfriend to avoid marriage to someone else. For some, the couple’s first or second sex together prompted ‘*Pai Khor’*. One girl eloped with her boyfriend after they had sex because she was worried that she might get pregnant; their elopement prompted their parents to discuss their marriage. Other motivations included avoiding the embarrassment of a possible premarital pregnancy, and desiring independence from parents.

None of the girls in this pathway were able to complete upper secondary education; most left school when their family or economic circumstances precluded them from continuing their studies (e.g., death of a parent; needed to work to help family earn income). In addition, five girls disliked school, two reported that the school was too far from their homes, and one believed that she was not good at studying. Two girls stopped attending school because they wanted to get married.

Half of the girls in this pathway experienced their sexual debut before union, while the other half experienced sexual debut after union (cohabitation, elopement, or marriage). For 14 girls, sexual debut was with the partner who got them pregnant; for many of these girls, first sex was described as consensual (girls reported wanting or agreeing to sex) or ‘planned/expected’ within union (girls did not mention if they wanted to have sex but implied that it was planned or expected because they were married/cohabitating/engaged). However, three girls described being pressured into sex (two before union, one within union), and for three other girls, first sex was forced by the partner who got them pregnant (two before union, one within union). Sexual debut often occurred in the context of ‘*Pai Len’*, usually with assurances from the boyfriend that he would take responsibility for any pregnancy through marriage:

*‘I spoke to my boyfriend that I am so afraid of getting pregnant but he said that there is nothing to worry about*. *If I have a baby*, *he will* Pai Khor.*’*

*(LA0201)*

One participant described that boyfriends expect that a girl should be willing to have sex a few months into the relationship:

*‘When having a boyfriend*, *you would normally chat for about 3–4 months*. *Like this guy*, *I knew him for four months*, *then we started having sex for the first time*.*’*

*(LA0202)*

Many girls in this pathway seemed to lack information about sex, pregnancy risk, and contraception. Less than half of the participants mentioned receiving any sex education at school; most reported receiving limited information about sex and contraception from friends, female relatives (e.g., mother, sister, aunt), and through observation (i.e., overhearing people in the community).

Eleven girls in this pathway did not intend to get pregnant (Pathway 4a), but one girl did not mind and two reported that their partner was happy about the pregnancy. Only one of the 11 girls reported using modern contraceptives (condoms) to delay first pregnancy, but condom use was inconsistent. The remaining 10 girls did not use any modern contraception method for a range of reasons: their partner objected/refused, they had never discussed contraception with their partners, they/their partner believed that pills could cause infertility or that contraceptives were not appropriate for married couples, or they underestimated their pregnancy risk. Six girls considered abortion, but most were convinced by their partner or parents to reconsider; one of these girls had a successful abortion with the support of her partner.

Four out of the 16 girls did not necessarily want to get pregnant but were aware that their partner wanted a baby (Pathway 4b). Three girls did not use any modern method of contraception because their partner wanted a baby; one girl used oral contraceptive pills and emergency contraceptive pills during engagement, but discontinued use when her partner expressed that he wanted to have a baby.

One girl in this pathway reported having an intended pregnancy (Pathway 4c) with the support of the husband’s parents; the couple did not use contraception for this reason. The girl described her first sexual experience with her boyfriend as forced, but the relationship then progressed consistent with Hmong traditional practices. Her boyfriend initiated elopement, after which they had a *Kan Mud Kaen* (‘tie-the-knot’) traditional ceremony, which is the way to socially announce that the couple would start a family together (but is not recognised as formal/legal marriage). The girl consented to this process and chose not to seek approval from her parents. The girl also shared that she pursued early union to lessen future financial burdens:

*‘[According to Hmong traditions] For those who are not married*, *they have to buy a cow for their parents when they die*. *… If we are married*, *we don’t have to buy [alone]*.*’*

*(LA0112)*

#### 5. Romantic relationship leading to parent or partner-led union and pregnancy

A second within-union pathway was characterised by romantic relationships that led to parent or partner-led union and unintended (Pathway 5a), partner-led (Pathway 5b), or intended (Pathway 5c) pregnancy. This pathway was observed across the three study areas and in urban, peri-urban, and rural settings. The fifteen girls in this pathway were of Khamu, Lao Loum, Lantan, Lue, Sila, and Yung ethnic backgrounds.

Unions were wanted and arranged mainly by parents, in some cases prompted by the girl’s boyfriend, due to reputational reasons (e.g., because the girl’s parents learned that the couple had premarital sex or the relationship was perceived as becoming serious and the parents wanted to avoid gossip); only three girls cited love as a motivation. Some girls were married off for economic security (to ensure financial provision for the girl or gain male labour for the household):

*‘My parents don’t have money to support my education and wanted me to get married to have good food*, *a place [to live]*, *and a family*, *like other people*.*’*

*(LA0229)*

In these settings, child marriage and early union was common and accepted in the community. Girls in this pathway had varying degrees of control over the decision to cohabitate or marry. Thirteen girls described having little or no real say in the decision once their parents had agreed to marriage or cohabitation, though in three cases, the couple was in-love. In one case, marriage was arranged but the girl had five months to get to know her partner before marriage and was more willing to marry:

*‘My boyfriend’s parents told me to marry and move in to help his family*. *They want to have additional daughter*. *At first*, *I did not like the idea*, *but I got married because I don’t want to disappoint them*. *But because he comes to my family so often*, *I started to change my mind that he is also good*. *Then*, *I felt it is okay to marry him*.*’*

*(LA0118)*

On the contrary, three other girls clearly expressed that they did not want to get married but were forced to. In one case, the girl’s boyfriend raped her, then informed her parents that they had sex, prompting the parents to accept his proposal to cohabitate.

All except three girls in this pathway experienced their sexual debut with the partner who got them pregnant, most before cohabitation or marriage. Four girls had sex for the first time within union and described sex as consensual or ‘planned/expected’ because they were married. For girls who experienced sexual debut outside of union, sex sometimes occurred in the context of ‘*Pai Len’* and special events, with boyfriends taking girls to guesthouses afterwards. Five girls described consenting/agreeing to engage in premarital sex after they had been drinking alcohol. Boyfriends’ promises to take responsibility for any pregnancy were important in convincing some girls to have sex. Three girls reported being raped by their boyfriend while drunk, of whom one was unconscious.

Almost all girls had some awareness about sex, condoms, and pills before pregnancy, though the extent of their understanding was unclear.

Eight girls and their partners did not intend to get pregnant (Pathway 5a), but in five cases, the partner was happy about/wanted to continue the pregnancy. Five of the eight girls did not use any modern contraception method because they were ambivalent about pregnancy, unaware of their contraception options, embarrassed to access pills, or discouraged by a health worker (e.g., because it ‘might impact [the girl’s] fertility’). Only three of the eight girls used modern methods (condoms, pills) to delay first pregnancy. The first girl used pills inconsistently and said she found it ‘annoying’ to access and use pills. The second girl used condoms but discontinued because a former boyfriend had told her that condom use could ‘hurt her womb’, and later discontinued taking pills after experiencing side effects. The third girl reported that she became pregnant while taking pills. Two of the eight girls considered abortion, but one was convinced by her mother-in-law to continue the pregnancy, while the other decided to continue the pregnancy because she was ‘afraid of sin’.

In three cases, the girl’s partner had made it clear they wanted a pregnancy following cohabitation or marriage (Pathway 5b), which influenced contraceptive use even when the girl did not want to get pregnant. The girls’ partners either refused to use condoms, or prohibited the girl from using contraceptives. One girl shared that her partner stopped using condoms after cohabitation; the girl took pills secretly, but her partner found out and disposed of the pills. Likewise, another girl recalled that her partner refused to use condoms after they moved in together. She wanted to try using pills, but no one would buy them for her, and she was too embarrassed to get them herself at the health centre. None of the girls considered abortion.

Four girls reported that her pregnancies were intended (Pathway 5c). The girl and her partner never used contraceptives because they planned to have a baby soon after they were married. Two girls did not access or use any contraceptives because they planned to get pregnant soon after union. The other two girls stopped taking contraceptive pills when the couple decided that they wanted to have a baby.

#### 6. No romantic relationship leading to parent or partner-led union and pregnancy

In the final pathway, three girls were compelled to marry a partner with whom they had no prior romantic relationship, and had a partner-led (Pathway 6a) or intended (Pathway 6b) pregnancy after they were married. This pathway was observed only in Luang Namtha, with one girl each residing in urban, peri-urban, and rural communities. Girls were of Khamu and Lamet ethnic backgrounds.

Girls in this pathway had little or no say in the decision to marry–marriage was arranged mainly by parents; in one case, the boyfriend facilitated betrothal by asking his parents to make a marriage proposal to the girl’s parents. All three girls (Lamet and Khamu ethnic backgrounds) only met their partner a few days before or on the day of their betrothal or wedding ceremony.

For two girls, marriage was arranged because their parents wanted it. For one of these girls, both sets of parents had agreed on the marriage long before the couple met, while the other girl met a man on Facebook, and he was eager to marry her. The girl’s father agreed to the union because he was concerned about rumours of the couple’s relationship. For the other girl, her parents arranged marriage because they could no longer afford to pay for the girl’s education, and the prospective husband was of good economic position. The girl also felt that studying was a ‘waste of time’ and that ‘being married is better’.

All girls in this pathway experienced their sexual debut after marriage. Two girls described their first sex as planned/expected because they were married, while one reported that her first sex with her husband was pressured. Girls had varied levels of knowledge about sex, reproduction, and contraception learned from school, female family members (e.g., mother, sister, aunt), and friends.

Two of the three girls did not want to get pregnant and experienced a partner-led pregnancy (Pathway 6a). They were taking contraceptive pills secretly, but their husbands found out and disposed of/hid them:

*‘I wanted to use condoms*, *but my husband did not want to*. *I bought pills and hid it from him*, *but he found it and was really upset*. *We got into a fight every time we talked about this*. *Then I got pregnant*.*’*

*(LA0225)*

The third girl reported that her pregnancy was intended, as her partner wanted to have a baby and her parents also encouraged her to have a baby while she was young (Pathway 6b). However, the girl had also heard misconceptions about contraceptives (e.g., that taking pills would ‘make the womb dry out’) and been forbidden by her husband to use contraceptives:

*‘I told him that I would use pills*. *But he said no*. *He said he wants to have a baby*.*’*

*(LA0219)*

In addition, her husband refused to use condoms as he believed that married couples should not use condoms.

## Discussion

Through qualitative investigation, this study aimed to identify pathways to adolescent pregnancy and important drivers within each pathway. Using visual timelines during interviews contextualised girls’ relationships and pregnancy in their life stories and facilitated understanding of the order of events. Through framework analysis, six pathways emerged, reflecting the narratives of 57 girls. These pathways to adolescent pregnancy highlighted common drivers that contributed to adolescent pregnancy.

### Barriers to SRH information and services, including contraception

Many girls in our study lacked accurate information about sex, reproduction, and contraception before sexual debut, as also observed in other research [29]. Some girls received information about sex and reproduction in school, but the most named friends and peers as their main sources of information. Currently, school-based comprehensive sexuality education (CSE)-life skills education (LSE) in Lao PDR is integrated into other subjects at both primary (e.g., moral education, science and environment) and secondary (e.g., biology, civic education, and safe use of social media) school levels [30]. CSE is mandatory in primary school but optional in secondary school, and coverage varies according to the type of school (e.g., public, private, faith-based) [30]. Some have noted gaps in the quality of teacher training and supervision, as well as learning materials (e.g., books, other materials), and that teachers did not feel confident delivering lessons on sexuality education [31].

At follow-up, participants elaborated that girls’ lack of knowledge about sex, reproduction, and contraception was sometimes because they did not think that the information they received in school was relevant to them (they felt these were ‘only for married people’). Girls often received more information from family members and healthcare providers about SRH (particularly contraception) after marriage or birth, suggesting that girls’ views that SRH information was ‘only for married people’ were being reinforced by social norms and structures. Prior studies in Lao PDR have found that parent-adolescent communication about SRH, relationships, and sex is low, with only one in five young people reporting ever discussing these topics with their parents [15, 17]. Our follow-up interview participants confirmed that some parents do not want to discuss sex with their adolescent daughters because they believe that they are ‘too young’. Negative cultural attitudes regarding sexual activity outside of marriage were also key contributors to young people’s reluctance to access SRH services and discuss SRH topics with healthcare providers [17]. Though there are no laws that explicitly prohibit young people from accessing SRH services [32], existing policies (e.g., 2019 National Sexual and Reproductive, Maternal, Newborn, Child and Adolescent Health Policy) lack specific guidance on parental consent requirements for minors to access contraceptives [33]. In the absence of laws or specific guidance, parental consent requirements are subject to healthcare providers’ discretion [33], and recent research suggests that healthcare providers could be limiting adolescents’ access to family planning services [34], especially since sex outside of marriage is socially unacceptable in Lao culture [12].

For many girls in our study, marriage was accompanied by expectations regarding contraceptive use and pregnancy. Hormonal contraceptives were accessed by some girls once they were in a union. However, side effects associated with these, or fears of side effects (particularly infertility), resulted in discontinuation of these methods. Couples underestimating their risk of pregnancy was also a salient theme across pathways resulting in unplanned pregnancies. These couples seemed less likely to use any contraception because they did not think they would get pregnant. Prior experiences of unprotected sex that did not result in pregnancy led to false confidence that unprotected sex would not result in a pregnancy, reflective of poor SRH knowledge. This was observed among participants who conceived before and after union.

Many girls in our study who had unplanned and partner-led pregnancies considered abortion and a few girls successfully undertook a termination. Even though abortion is illegal in Lao PDR except to save a woman’s life or to protect a woman’s physical health [28], recent data indicate that a drop in contraceptive use during the COVID-19 pandemic was associated with an increase in the unsafe abortion rate [35]. Our findings suggest that despite its illegality, abortion remains common in Lao PDR and women–including adolescent girls–need access to safe abortion services.

### Partner’s control over reproductive decision-making

Partner’s control over reproductive decision making (including contraceptive use and desire for a pregnancy) was another key driver across pathways. For pathways in which the couple jointly did not plan pregnancy, condom use was usually discontinued when the boyfriend decided to stop using and instead, promised to take responsibility for any pregnancy. Some couples, but particularly males, reportedly viewed condoms as inappropriate for a couple in union. During follow-ups, participants elaborated that married couples found to be using condoms may invite suspicion, because condom use was associated with sexually transmitted infection or marital infidelity. This aligns with other research where male participants expressed that they did not like condoms and felt that condoms were for men who were cheating on their wives [36]. Follow-up participants added that girls often felt they had no choice but to accept the partner’s preference. This is consistent with the findings of other studies in Lao PDR, that unmarried young women usually deferred to their male partner when it came deciding whether or not to buy and use contraceptives [18, 19], even when the young woman’s preference differed from that of their partner [17].

Some participants experienced a partner-led pregnancy despite being aware of contraceptive methods and, in some cases, having attempted to use them. In these cases, the partner/husband wanted to have a baby, but in some cases, family members also encouraged pregnancy soon after union. A study in Savannakhet Province found that some women accepted their partner’s decision on contraception (including not using any), although some women from urban areas reported that they would try to negotiate or covertly use contraceptives if they disagreed with their partner’s decision [36]. Some of our follow-up interview participants believed that married women had more say in timing of subsequent pregnancies and contraceptive use after they had already had one baby; more so if the baby was a boy. Yet, we noted a few cases of married girls attempting to use contraceptives covertly, indicative of unequal power in their relationships [37].

### Male sexual entitlement and alcohol use driving and pressured/forced sex

Experiences of pressured and forced sex were observed across pathways, usually involving alcohol, and some of which were soon followed by pregnancy. Alcohol consumption is common among Lao young people, especially in rural settings [38]. In our study, alcohol consumption combined with notions of male sexual entitlement contributed to girls’ risk of pressured and forced sex as some boys/men took advantage of girls under the influence of alcohol. However, in contrast to findings from related research in Indonesia [39], among the subset of girls who experienced forced sex outside of union, it was more common for girls to not marry their assailant. Girls becoming pregnant due to forced sex outside of union represented their own pathway to adolescent pregnancy, and these participants either refused to continue a relationship with the partner who got her pregnant, or her parents/partner decided against pursuing or continuing a union. Alongside interventions seeking to engage men and boys in eliminating violence against women and girls, there is an urgent need for gender transformative programmes that promote respect in relationships and address harmful use of alcohol and other drugs.

### Cultural acceptance of child marriage and early union

Consistent with prior research [18], community acceptance of child marriage and early union was an important driver of adolescent pregnancy in this study. Our findings indicate that child marriage and early union is viewed as the most acceptable response to adolescent pregnancy outside union. This was consistent with other research in rural communities in Lao PDR that found that young people, parents, and community elders had favourable attitudes and norms regarding child marriage and adolescent pregnancy as it was the norm in these settings [18]. Most of our participants experienced sexual debut before cohabitation or marriage, often following assurance from the boyfriend that they would take responsibility for any pregnancy through ‘*Pai Khor*’. When an unmarried girl in a romantic relationship became pregnant, parents expected the couple to marry. Betrothal through ‘*Pai Khor*’ was crucial as it served to lessen the negative social consequences for the pregnant girl and her family. Notably, for a few girls who had unplanned pregnancies, there was less urgency for marriage to happen before the birth of the baby–some parents accepted cohabitation with the promise of marriage later. This is consistent with another study that found that among women aged 20–24 years who have ever been in a union and conceived before union, only 58% of women from Laos were married by the time they gave birth [14].

In some cases, girls acknowledged that it was common in their community or village for adolescent girls to marry and begin childbearing, and some stopped attending school by choice once they entered a union. Similar findings were noted in Indonesia, where adolescents viewed becoming a wife and mother as a valued and realistic life goal, particularly when there were constraints to education and work [40]. Cohabitation or marriage both served as an indication of a couple’s long-term commitment to each other. However, during follow-up interviews, participants clarified that though cohabitation was common, girls in cohabiting unions still aspired to be formally married. Other research has found that it is common for Lao girls to aspire for marriage and childbearing in adolescence, with some young women expressing apprehension that they may not be able to attract a suitable partner if they wait until age 20 to marry [18].

For a smaller number of participants, particularly in Luang Namtha, girls were pressured into marriage by parents for economic reasons, and this was soon followed by pregnancy. Some parents expected or pushed for their daughter to cohabitate or marry to ensure financial security for the girl by marrying her into a family with a better economic position. In other cases, parents (and some girls) saw marriage as a way to ease the financial burden on the family, or to gain an additional labourer to help the farming household. For these participants, parents’ expectations of marriage remained a primary driver of adolescent pregnancy.

Follow-up interview participants elaborated that if a married couple does not have a baby soon after marriage, they may be mocked, ridiculed, or suspected of ‘something wrong’–such as the man having ‘weak sperm’ or the woman having ‘too much sex’ or using contraceptives ‘too much/for too long.’ This was indicative of the social pressure experienced by both females and males to prove their fertility once in a union, and illustrates how child marriage and early union norms within the community are contributing to adolescent pregnancy.

### Attitudes and norms regarding sex and pregnancy outside of union

In almost half of our sample, premarital sex and avoiding the risk or shame of a pregnancy outside of union served as drivers of child marriage and early union, which then facilitated adolescent pregnancy. For some girls, the discovery by family that they had engaged in premarital sex prompted some boyfriends to propose or parents to arrange (or consent to) their daughter’s engagement to avoid embarrassment or having to pay village fines. In our study, about half of participants in the post-union pathways and most of the participants in the pre-union pathways experienced sexual debut before union, indicating discordance between social ideals disapproving of premarital sex and young people’s lived realities. This aligns with similar research from the Philippines noting that even amid social disapproval of premarital sex, many young people engage in sexual relations before cohabitation or marriage [41, 42]. Others have noted that while premarital sex is stigmatised and not socially accepted in Lao culture [12, 17, 34], in certain areas (i.e., rural, and rural-off-road) and ethnic minority communities, traditional values, beliefs, and practices are more permissive regarding adolescent premarital sexual relations [10, 18, 43].

### Strengths and limitations

We note some limitations of our study. First, we used a combination of purposive and snowball sampling in two provinces in Lao PDR to recruit young women who were best placed to address our research topic. As such, our findings are not necessarily representative of adolescents’ pathways to pregnancy in other provinces, regions, or the country. However, we captured diverse contexts and crosscutting factors that will be important to explore with girls from other geographical areas, and socioeconomic and ethnolinguistic backgrounds.

Second, and related to our sampling and study areas, we were able to collect rich qualitative data through our in-depth timeline interviews. While we were able to gather the depth of information that we needed to address our research objectives, given our focus on strands within individual narratives [44], it is possible that other pathways featuring novel contextual and crosscutting factors may still eventually emerge if more data were to be continuously collected [45]. Nevertheless, our study was able to identify eight diverse pathways based on key life events, motivations, and decision-making related to sexual relations, contraceptive use, non-use and discontinuation, pregnancy, and union formation, all of which would likely still be important in any alternative pathways to adolescent pregnancy.

We conducted follow-up interviews to validate and clarify our study findings with a selected number of participants from the individual interviews. We opted to conduct follow-up interviews over the phone due to prevailing COVID-19 restrictions and to minimise exposure risk for our participants and Lao PDR research team. It is possible that conducting the follow-up activity through in-person, participatory group discussions may have generated different (e.g., more in-depth, detailed) feedback from our participants, as participants would have had the opportunity to engage with each other and elaborate on their ideas, which was not possible through one-on-one follow-up interviews over the phone. Yet, the follow-up interviews validated and clarified our findings and interpretations, particularly regarding social pressure for couples to begin childbearing soon after child marriage and early union, and the common reasons for male unwillingness to use condoms. These will be important considerations for future research as well as programmes and policy.

### Implications for research and practice

Our findings contribute valuable insights regarding the different ways that adolescent girls in Lao PDR navigate romantic relationships, sex, and union in relation to their experiences of adolescent pregnancy.

Our study found that many adolescent pregnancies in Lao PDR occur outside of formal marriage despite social disapproval of premarital sex and pregnancy. Adolescent girls’ experiences of sex and pregnancy were affected by a lack of knowledge about SRH, power imbalances with their partners, and girls’ lack of agency over if and when to have sex, use contraceptives, and begin childbearing. These were also occurring within sociocultural contexts where parents, community members, and adolescent girls themselves viewed child marriage and early union as an acceptable alternative to education (especially when the girl’s family faced financial hardship), and as the most acceptable response to a pregnancy outside of union.

Moving forward, it will be imperative to address adolescent girls’ barriers to SRH information and services, including contraception. This requires strengthening comprehensive sexuality education (CSE)/life skills education (LSE) both within and outside school settings by increasing young people’s access to CSE/LSE, but also by improving the content covered and the quality of delivery. CSE/LSE content should include a strong focus on addressing myths about contraception, equipping girls with the skills (e.g., communication, negotiation, consent) they need to make informed decisions and assert themselves in their relationships, teaching young people to engage in respectful relationships, and engaging girls and boys to critically analyse harmful gender norms. Further implementation research is needed to understand how to effectively deliver CSE curriculum at scale, and how to effectively address harmful gender norms.

Alongside this, we recommend ensuring that girls have access to non-judgemental, adolescent-responsive healthcare, and increasing availability of and improving access to affordable (or free) contraception. SRH service providers will need to support all girls (whether in a union or not) to access and use their chosen contraceptive method. This would need to be complemented by initiatives to transform parents’, community members’, and healthcare workers’ perceptions that SRH information and services are not appropriate for unmarried adolescents, including addressing individual and social attitudes that contribute to male resistance to condom use.

The acceptance of child marriage and early union–as well as adolescent pregnancy–is deeply rooted in Lao customs, traditions, values, and beliefs, including the view that associates womanhood with being a wife and mother [18]. Culturally responsive approaches that foster adolescent girls’ sexual and reproductive agency will be required to shift these, and it will be important to ensure girls have the resources they need to pursue their educational and work goals. At the community and societal levels, this requires addressing sociocultural and financial drivers of child marriage/early union and adolescent pregnancy, by working toward shifting notions of child marriage and early union as a ‘solution’ for premarital pregnancy, and helping families build economic resilience as a preventive measure against child marriage/early union and adolescent pregnancy.

## Supporting information

S1 Checklist Inclusivity in global research. (DOCX)
